# The cloacal microbiome of a cavity-nesting raptor, the lesser kestrel (*Falco naumanni*)

**DOI:** 10.7717/peerj.13927

**Published:** 2022-10-06

**Authors:** Alessandra Costanzo, Roberto Ambrosini, Andrea Franzetti, Andrea Romano, Jacopo G. Cecere, Michelangelo Morganti, Diego Rubolini, Isabella Gandolfi

**Affiliations:** 1 Department of Environmental Sciences and Policy, University of Milan, Milan, Italy; 2 Department of Earth and Environmental Sciences, University of Milan—Bicocca, Milan, Italy; 3 Area Avifauna Migratrice, Istituto Superiore per La Protezione e La Ricerca Ambientale (ISPRA), Ozzano Emilia, (BO), Italy; 4 IRSA-CNR, Water Research Institute-National Research Council of Italy, Brugherio, Italy

**Keywords:** ASV, Lesser kestrel, Microbiome, Wild raptor, *Falco naumanni*, Cavity-nesting bird

## Abstract

**Background:**

Microbial communities are found on any part of animal bodies exposed to the environment, and are particularly prominent in the gut, where they play such a major role in the host metabolism and physiology to be considered a “second genome”. These communities, collectively known as “microbiome”, are well studied in humans and model species, while studies on wild animals have lagged behind. This is unfortunate, as different studies suggested the central role of the gut microbiome in shaping the evolutionary trajectories of species and their population dynamics. Among bird species, only few descriptions of raptor gut microbiomes are available, and mainly carried out on captive individuals.

**Objectives:**

In this study, we aimed at improving the knowledge of raptor microbiomes by providing the first description of the gut microbiome of the lesser kestrel (*Falco naumanni*), a cavity-nesting raptor.

**Results:**

The gut microbiome of the lesser kestrel was dominated by Actinobacteria (83.9%), Proteobacteria (8.6%) and Firmicutes (4.3%). We detected no differences in microbiome composition between males and females. Furthermore, the general composition of the microbiome appears similar to that of phylogenetically distant cavity-nesting species.

**Conclusions:**

Our results broaden the knowledge of raptor gut microbial communities and let us hypothesize that the distinct nest environment in terms of microclimate and presence of organic material from previous breeding attempts, to which cavity-nesting species that reuse the nest are exposed, might be an important driver shaping microbiomes.

## Introduction

Microbial communities, collectively known as the “microbiota”, can be found on all the surfaces of macroorganisms exposed to the environment, such as the skin, gills, feathers and in the cavities of the body, particularly in the gut (Colston & Jackson, 2016). In particular, many studies carried out so far have focused on the composition and functional characteristics of the mammalian gut microbiota and on their genes (the so-called “microbiome”), which provide such a large contribution to the host metabolism to be considered a “second genome” for the host (Zhu, Wang & Li, 2010). The gut microbiota can be viewed as an additional organ that performs functions that are not coded by the host genome. For instance, the gut bacteria are pivotal in enhancing the degradation of dietary polysaccharides, protect the host against pathogenic infections, and are involved in several aspects of intestinal development (Hooper & Macpherson, 2010). Different studies are also supporting the idea that, on evolutionary time scales, the gut microbiota has established important interconnections with the central nervous system (the so-called microbiota–gut–brain axis), with profound influences on host behavior and stress response (Rhee, Pothoulakis & Mayer, 2009; Forsythe, Kunze & Bienenstock, 2012; Daisley et al., 2022). The gut microbiota is important for organismal functioning, and the identification of a reference microbiota for a species is pivotal for the assessment of any deviation associated with a disease state (Clemente et al., 2012) and for evolutionary analyses (Hird, 2017). In recent years, the microbiome of many organisms has been sequenced, with special focus on model species, livestock and wildlife species at risk of extinction (Bahrndorff et al., 2016; Pascoe et al., 2017). Unfortunately, studies on avian microbiomes have lagged behind, and even if the study of gut microbiomes of wild species is rapidly growing, there is still a large gap of knowledge even in the basic description of the gut microbial communities. This gap limits our ability to understand the ecological and evolutionary implications of the ‘second genome’ of birds (Bodawatta et al., 2022). There is in fact a growing interest in understanding the factors that affect the microbiota of animals, considering its importance, for instance, in shaping evolutionary trajectories (Ezenwa et al., 2012). For example, a host-microbiota coevolution might explain the resistance of scavenging vultures, which mainly feed on carcasses, to toxins (Roggenbuck et al., 2014). Indeed, the microbiota not only exploits the flow of protein-rich food assumed by the hosts, but also provides resistance to pathogens and contributes to digestion (Roggenbuck et al., 2014). The study of the microbiota might also have potential implications in conservation biology (Bahrndorff et al., 2016; Bodawatta et al., 2022); as an example, an increase in pesticide exposure might mediate the decline of many bird species, especially those associated with agroecosystems, by altering hosts’ microbial communities (Daisley et al., 2022).

Therefore, despite what we already know about avian microbiomes, there are still many open questions which would deserve studies on microbiomes to be addressed using a more diverse set of avian taxa. In fact, some orders still appear underrepresented in gut microbiome studies. In particular, raptors (orders Accipitriformes, Falconiformes and Strigiformes) are poorly represented. Indeed, to date, very few descriptions of raptor gut microbiomes have been published (Colston & Jackson, 2016; Nagai et al., 2019; Guan et al., 2020; Oliveira et al., 2020; Song et al., 2020; Zhou et al., 2020), mainly carried out on animals reared in captivity or admitted to wildlife rescue centers. However, it is likely that the gut microbiome of those animals was altered due to illnesses, trauma, medications, and/or captive rearing conditions (Oliveira et al., 2020) and, as a consequence, that their microbiome differs as compared to that of their wild conspecifics (Trevelline et al., 2019). Here, we improved the knowledge of raptor microbiomes by reporting the first description of the cloacal microbiome of the lesser kestrel (*Falco naumanni*). In particular, we focused on the microbiome of the most distal part of the gut, the cloaca, as it can be easily collected by means of a swab and does not require extensive handling or retention of the animal and, despite not fully representing the gut microbiome, it is considered a good proxy of it (Bodawatta et al., 2020). We subsequently performed a qualitative comparison between the microbiome of the lesser kestrel and that of other bird species based on different factors that might shape the microbiome composition such as phylogeny, diet and nesting habits. Finally, we also aimed at investigating the potential differences in the cloacal microbiome among sexes because such difference has been reported to occur in diverse bird species (Ambrosini et al., 2019; Corl et al., 2020).

## Methods

### Study species and field procedures

The lesser kestrel is a small (ca. 120 g), sexually dimorphic, cavity-nesting falcon (Cramp, 1998). It reproduces in colonies ranging in size from a few pairs to several thousand individuals, which often settle in urban habitats (Negro et al., 2020). European populations breed mainly in the Mediterranean region (south of 45°N) and migrate to sub-Saharan Africa (chiefly Sahelian savannahs) during the non-breeding period (Sarà et al., 2019). The study was conducted during April–July 2017 in Matera (southern Italy, 40°67′N, 16°60′E), which hosts one of the largest lesser kestrel colonies worldwide (ca. 1,000 breeding pairs, La Gioia, Luca & Lorenzo, 2017). Here, lesser kestrels breed in holes and crevices of buildings in the old town, but many pairs settle in specially designed nestboxes placed on the terraces of large public buildings (Podofillini et al., 2018; Podofillini et al., 2019; Morinay et al., 2021). Birds forage in the farmland areas surrounding the city (Cecere et al., 2018), hunting on grasslands and cereal crops (Morganti et al., 2021) and feeding mainly on large invertebrates, lizards and small mammals. Adult birds were captured by hand within their nestbox during the pre-breeding stage (*i.e.,* before egg laying) according to well-established protocols aimed at minimizing disturbance (see *Animal welfare note* below). Cloacal microbiomes were collected using sterile DNA-free microbiological nylon dry swabs (minitip FLOQSwabs 516CS01, COPAN, Brescia, Italy). All samples were kept at +4 °C while in the field and subsequently maintained at −20 °C until processing (Musitelli et al., 2018a; Musitelli et al., 2018b). We sampled cloacal microbiomes from four females and five males.

### DNA extraction and sequencing

Total DNA was extracted from the swabs using Fast DNA Spin kit for Soil (MP Biomedicals, Solon, OH, USA) according to manufacturer’s instructions. The V5–V6 hypervariable regions of the bacterial 16S rRNA gene were PCR-amplified with barcoded primers to allow sample pooling and sequence sorting. Amplicons were sequenced by MiSeq Illumina (Illumina, Inc., San Diego, CA, USA) with a 2 × 300 bp paired-end protocol. Sequencing was carried out at Consorzio per il centro di Biomedicina Molecolare (CBM) (Trieste, Italy). Amplicon Sequence Variants (ASVs) were inferred using the DADA2 pipeline (Callahan et al., 2016), with trimming of the forward and reverse reads after 180 and 150 bases, respectively, and quality filtering with a maximum number of expected errors equal to 0.5 per read. The two reads were therefore merged and taxonomically assigned with the stand-alone version of the RDP classifier. Further details on DNA extraction from the swabs and Illumina sequencing are reported in Ambrosini et al. (2019). Raw sequences, together with the code and pipeline used, are available as Supplementary information.

### Animal welfare note

Capture and handling were conducted by the Italian Institute for Environmental Protection and Research (ISPRA), under the authorization of Law 157/1992 (Art. 4(1) and Art. 7(5)), which regulates activities on wild birds and mammals in Italy. All procedures adhered to established guidelines for the treatment of animals in behavioural research (ASAB/ABS, 2020). The sampling of cloacal microbiomes is minimally invasive and involves gently inserting a small sterile swab in the cloaca, then rotating it for ca. 30 s. Cloacal microbiomes were sampled immediately after capture. Birds were then individually marked by applying a unique metal ring or identified (if ringed in previous years) and released in their nestbox within 5 min from capture.

### Statistical analyses

Coverage of each sample was evaluated by the Good’s method (Good, 1953). Alpha diversity of each sample was estimated by the means of three different indices, namely: number of ASVs; Shannon diversity index, which represents the diversity of a population (Shannon, 1948); and Gini index, which ranges between 0 and 1 and is a measure of the evenness of a community (Gini, 1912), with lower values indicating more evenness. The three indices were subsequently compared between sexes by t-tests. These alpha diversity indices were calculated on a sample of sequences rarefied to 1,780 (slightly less than the minimum number of sequences in a sample), while beta diversity analysis was carried out on the non-rarefied dataset (Ambrosini et al., 2019). The homogeneity of the multivariate variance between sexes was evaluated by using the betadisper function in the R package Vegan (Borcard, Gillet & Legendre, 2011). Differences in the structure of the bacterial communities of males and females were further investigated by a redundancy analysis (RDA). All the multivariate analyses were performed by using the Hellinger-transformed ASV abundances (De Cáceres, Legendre & Moretti, 2010; Legendre & Legendre, 2012), in order to decrease the importance of ASV abundance over occurrence and avoid the double-zero problem when comparing ASV composition between samples (Musitelli et al., 2018a; Musitelli et al., 2018b; Ambrosini et al., 2019). Analyses were performed in R 3.6.2 (R Core Team, 2016) with the Vegan (Oksanen et al., 2019) and Multtest (Pollard, Dudoit & van der Laan, 2005) packages.

## Results

A total of 107,962 sequences were obtained from the nine sampled individuals. Good’s index for the whole dataset was ≥ 0.998, indicating a good coverage of our libraries, which was further confirmed by rarefaction curves (Fig. S1). Subsequently, 1,374 (1.27%) sequences were removed from the dataset for all the analyses because they were identified as chloroplasts (7, 0.01%), unclassified at domain level (1,327, 1.23%) or global singletons (*i.e.,* sequences appearing only once in the whole database) (40, 0.04%). After this selection, the number of sequences per sample ranged between 1,787 and 32,183.

Identified bacteria belonged to a total of 19 phyla, 103 families and 135 genera. Abundant phyla were Actinobacteria (83.9%), Proteobacteria (8.6%) and Firmicutes (4.3%). Abundant families were Corynebacteriaceae (81.8%, phylum Actinobacteria) and Enterobacteriaceae (2.6%, phylum Proteobacteria) that were present in all samples, followed by Helicobacteraceae (2.5%, phylum Proteobacteria). *Corynebacterium* (81.3%, phylum Actinobacteria) was the most abundant genus. *Helicobacter* and *Escherichia/Shigella* were the second (2.5%, phylum Proteobacteria) and the third (1.9%, phylum Proteobacteria) most abundant genera respectively, followed by *Streptobacillus* (1.3%, phylum Fusobacteria) and *Enterococcus* (1.2%, phylum Firmicutes).

**Figure 1:**
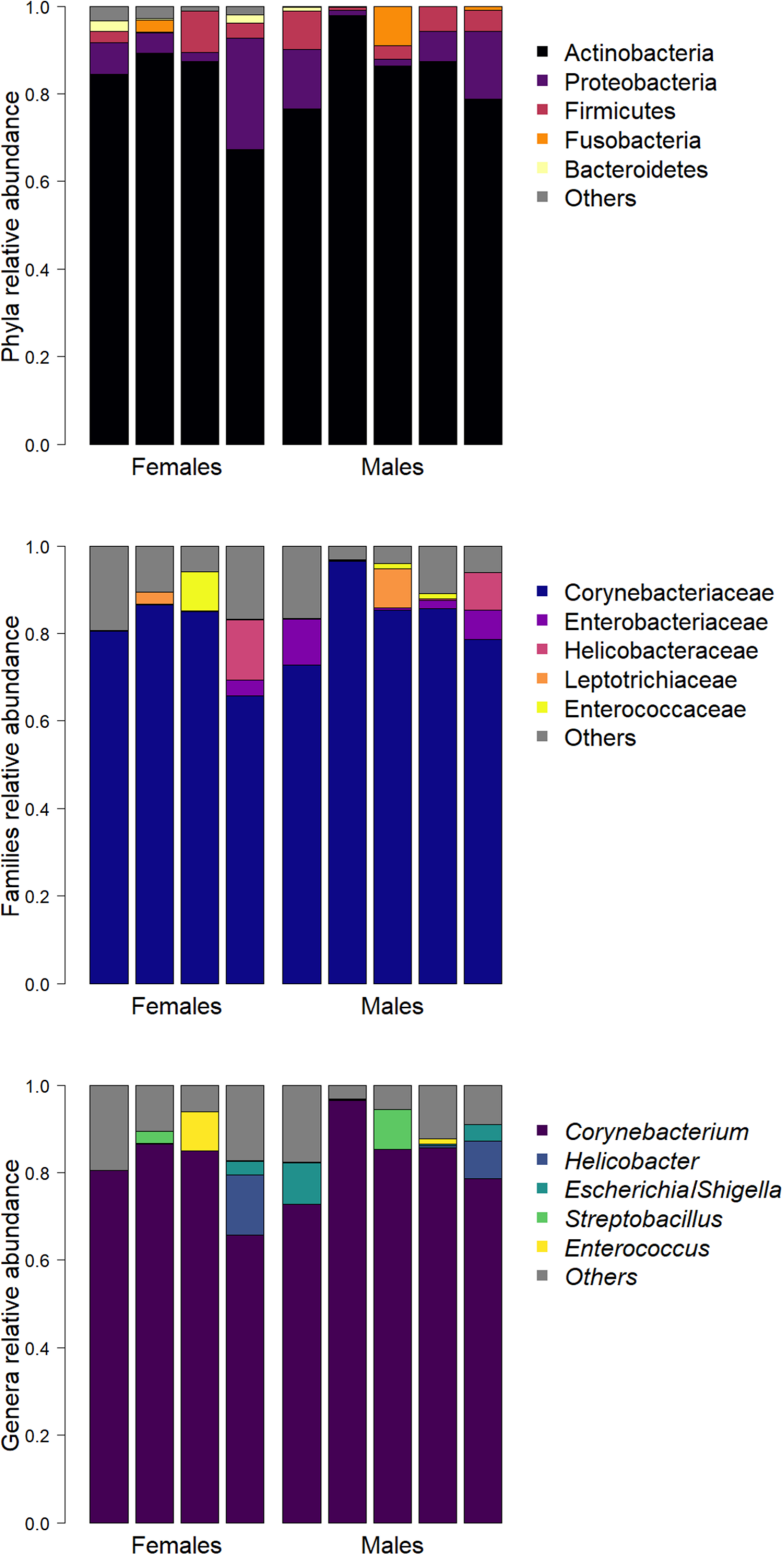
Barplots of the relative abundance of the five most represented phyla (A), families (B), and genera (C) in each sample.

Only one ASV (genus *Corynebacterium*) was present in all samples, seven (1.38%) ASVs were present in more than 50% of samples, and 403 (79.49%) were found in only one sample. The mean relative abundances of the 5 most represented phyla, families and genera per sample are reported in Fig. 1.

ASV richness did not differ between sexes (t_7_ = 1.63, *P* = 0.15), with an average value (± s.d.) of 52.5 ± 19.4 in females and 30.8 ± 21.3 in males. The diversity indices of male and female bacterial communities were also similar (Shannon diversity index, t_7_ = 0.80, *P* = 0.45; Gini index, t_7_ = 1.26, *P* = 0.25). The multivariate variance of the cloacal bacterial community was homogeneous between the sexes (*F*_1,7_ = 0.1, *P* = 0.75) (Fig. S2), and the bacterial communities of males and females did not differ (RDA: *F*_1,7_ = 1.22, *P* = 0.22).

## Discussion

The lesser kestrel cloacal microbiome was found to be dominated by the phyla Actinobacteria, Proteobacteria and Firmicutes, while Bacteroidetes and Fusobacteria were represented in a lower proportion. The presence of those phyla is not surprising, as they represent a conserved consortium within the gut, shared by most vertebrates (Ley et al., 2008b; Waite & Taylor, 2014; Hird et al., 2015). Nevertheless, the lesser kestrel cloacal microbiome harbours a larger relative abundance of Actinobacteria and a lower one of Firmicutes compared to the other raptor species that were sampled both in captivity and in natural conditions. Indeed, the most represented phyla among all the raptor species for which a microbiome description is available were Firmicutes, Proteobacteria and Bacteroidetes (Nagai et al., 2019; Oliveira et al., 2020; Zhou et al., 2020). Although some of these latter studies were performed on fecal microbiomes, qualitative similarities among the microbiomes of raptors were expected because of several potential, non-mutually exclusive, mechanisms related to phylogeny, diet and nesting habits. In fact, according to the hypothesis of a phylosymbiosis effect on the structure of microbiomes (Ochman et al., 2010; Brooks et al., 2016; Youngblut et al., 2019; Song et al., 2020), at least a similarity in the gut microbiome of the lesser kestrel and of that of a closely related species, the Eurasian kestrel (*Falco tinnunculus*), was expected, although the studies on the Eurasian kestrel were performed on fecal samples (Guan et al., 2020; Zhou et al., 2020). However, we found that the Eurasian kestrel microbiome is characterized by a high abundance of Proteobacteria (76.38%), followed by Firmicutes, Bacteroidetes and Actinobacteria (Guan et al., 2020; Zhou et al., 2020). Moreover, it is important to remember that most previous descriptions of raptor microbiomes were performed on captive animals, and captivity can deeply alter the gut microbiome structure (Wienemann et al., 2011; San Juan, Castro & Dhami, 2021); therefore, any comparison between the lesser kestrel microbiome described in this work and the few previous studies on different raptor species should be made with this caveat in mind. Another factor that might potentially play a critical role in determining similarities and differences among the gut microbiomes of different species is the foraging habits. Indeed, several previous studies highlighted a convergence of bacterial communities in species sharing the same diet (Ley et al., 2008b; Ley et al., 2008a; Karasov, Rio & Caviedes-Vidal, 2011). In mainly insectivorous species, such as the lesser kestrel, a strong energetic cost is imposed by the indigestibility of the energy-rich carbohydrate chitin; in vertebrates, chitinolytic activity originates from endogenous enzymes, but can also be enhanced by enzymes produced by gut microorganisms (Šimůnek et al., 2001). It was therefore possible to envisage a convergence between the microbiomes of the lesser kestrel and of the oriental honey buzzard (*Pernis ptilorhynchus*), a medium-sized raptor that mainly feeds on adults, larvae, pupae and combs of social hymenopterans (Nagai et al., 2019). However, the gut microbiota of the oriental honey buzzard was characterized by a high abundance of Firmicutes (and not of Actinobacteria as in the lesser kestrel), such as other carnivorous raptors (Oliveira et al., 2020). Finally, we speculated that the nest environment might contribute to the shaping of the gut microbiome. Indeed, previous studies have reported a larger contribution of the horizontal acquisition of microbiome from the surrounding environment over the vertical transmission from parents, despite it is sometimes difficult to discern the two transmission modes (Bright & Bulgheresi, 2010; van Veelen, Salles & Tieleman, 2017; Roughgarden, 2020). In particular, nest environment seems to contribute to the shaping of the gut microbiome (Lucas & Heeb, 2005; Teyssier et al., 2018), as stressed by the “nidobiome” concept (Campos-Cerda & Bohannan, 2020). Nest architecture, for instance, might influence the richness of the bacterial communities. Cavity nests offer the most suitable conditions for bacterial growth given their stable microclimatic conditions (Mehmke et al., 1992; Singleton & Harper, 1998; Goyache et al., 2003; Gonzalez-Braojos et al., 2012); in addition, nest reuse is widespread among cavity-nesting birds and favours bacterial growth between consecutive breeding seasons (Mazgajski, 2007). The lesser kestrel is a cavity nester that lays its eggs directly on the substrate without lining the nest nor adding specific aromatic plants useful to decrease the nest bacterial loads before egg laying (Mennerat et al., 2009). It also prefers nests containing organic material from previous breeding attempts (Podofillini et al., 2018; Morinay et al., 2021). Both males and females spend time in the nest, incubating the eggs and attending the offspring (Ramellini, 2022; Sarasola, Grande & Negro, 2018). Thus, the microbial community present in the nest cavity could be transferred in the digestive system during preening. The nest microbiome might therefore affect the microbial assembly of the lesser kestrel gut microbiome, potentially modifying and masking any association between the microbiome and the bird’s diet or phylogeny. If this was the case, it should be possible to observe similarities among the microbiomes of the lesser kestrel and those of other, even non-raptor, birds, which have similar nesting habits. Interestingly, we noted a similarity between microbiomes of the lesser kestrel and of the tree swallow (*Tachycineta bicolor*), a small passerine bird that nests in cavities (Hernandez et al., 2020). In particular, both species showed a predominance of Actinobacteria and Corynebacteriaceae at the phylum and at the family level, respectively. In addition, the only bacterial genus present in 100% of samples in both species belongs to the genus *Corynebacterium.* Despite containing some pathogenic species (Katsukawa et al., 2016; Bratcher, 2018), *Corynebacterium* spp*.* are frequently isolated from healthy birds showing different nesting habits (Bangert et al., 1988; Fernández-Garayzábal et al., 2003; Goyache et al., 2003; Risely et al., 2018) and are thought to positively influence the host immune response and fat storage (Zhang, Yang & Zhu, 2021). However, it should also be noted that the tree swallow is an insectivorous species; therefore, we cannot exclude a synergistic effect of the generalist insectivorous diet and the nesting habit in determining the observed microbiome similarities between the two species. Future studies should therefore be aimed at discerning between these two potential sources of variation, by investigating the relative importance of the diet-driven selection on gut microbiome *vs.* the potential contribution from the microbiome of the nest cavity in shaping the host microbiome.

In this study, we also found no difference in alpha- and beta-diversity of cloacal bacterial communities between males and females. Despite that our results should be taken with caution due to the small sample size, they are consistent with the observation of lack of sex-related differences in other bird species (Hernandez et al., 2020; Bodawatta et al., 2021). However, in some species, males and females were found to host different bacterial communities (Ambrosini et al., 2019; Corl et al., 2020). This inconsistency among studies might be related to differences among host species in the physiology, reproductive state, and fitness traits of the two sexes that may (or may not) differentially modulate the gut microbiome. However, these hypotheses cannot be easily disentangled, and a larger sample size, possibly in the frame of ad-hoc studies, is needed to confirm the lack of differences in gut microbiome composition between sexes.

In conclusion, this study provided the first description of the lesser kestrel cloacal microbiome, highlighting a lack of similarities between the microbiome of our model species and that of other raptors. However, a comparison with a phylogenetically distant species led us to suggest that, for cavity-nesting species that re-use the nest in different breeding seasons, the nest environment might play a role in shaping the composition of the gut microbiome, thus supporting the so-called “nidobiome” concept (Campos-Cerda & Bohannan, 2020). Future experimental studies should be carried out to disentangle the factors shaping the gut microbiome of the lesser kestrel, and to understand the impact of host’s life-history traits, living habitat (*e.g.*, urban *vs* rural birds), and physiology on gut microbiome.

##  Supplemental Information

10.7717/peerj.13927/supp-1 Supplemental Information 1 Rarefaction curves for the 9 cloacal microbiome samples

10.7717/peerj.13927/supp-2 Supplemental Information 2 Biplot from the Principal Component Analysis on Hellinger-transformed ASV abundance. Orange circles denote females, blue squares represent males. Polygons of the same color include the cloacal microbiomes of individuals in the same group. Variance explain

10.7717/peerj.13927/supp-3 Supplemental Information 3 Asv and sample information

10.7717/peerj.13927/supp-4 Supplemental Information 4 Code and pipeline

10.7717/peerj.13927/supp-5 Supplemental Information 5 Fastq sequences 1

10.7717/peerj.13927/supp-6 Supplemental Information 6 Fastq sequences 2

10.7717/peerj.13927/supp-7 Supplemental Information 7 Fastq sequences 3

10.7717/peerj.13927/supp-8 Supplemental Information 8 Fastq sequences 4

10.7717/peerj.13927/supp-9 Supplemental Information 9 Fastq sequences 5

10.7717/peerj.13927/supp-10 Supplemental Information 10 Fastq sequences 6

10.7717/peerj.13927/supp-11 Supplemental Information 11 Fastq sequences 7

10.7717/peerj.13927/supp-12 Supplemental Information 12 Fastq sequences 8

10.7717/peerj.13927/supp-13 Supplemental Information 13 Fastq sequences 9

10.7717/peerj.13927/supp-14 Supplemental Information 14 Fastq sequences 10

10.7717/peerj.13927/supp-15 Supplemental Information 15 Fastq sequences 11

10.7717/peerj.13927/supp-16 Supplemental Information 16 Fastq sequences 12

10.7717/peerj.13927/supp-17 Supplemental Information 17 Fastq sequences 13

10.7717/peerj.13927/supp-18 Supplemental Information 18 Fastq sequences 14

10.7717/peerj.13927/supp-19 Supplemental Information 19 Fastq sequences 15

10.7717/peerj.13927/supp-20 Supplemental Information 20 Fastq sequences 16

10.7717/peerj.13927/supp-21 Supplemental Information 21 Fastq sequences 17

10.7717/peerj.13927/supp-22 Supplemental Information 22 Fastq sequences 18

## Additional Information and Declarations

### DNA Deposition

The following information was supplied regarding the deposition of DNA sequences: Raw sequencing data are available at the NCBI-SRA (Sequence Read Archive), with BioProject ID PRJNA816317 (BioSample accessions SAMN26669086–SAMN26669094). The sequences are also available as Supplemental Files.

### Data Availability

The following information was supplied regarding data availability: Raw data are available in the Supplemental Files.
