# Supplementary figures and images for: The cloacal microbiome of a cavity-nesting raptor, the lesser kestrel (Falco naumanni)
Source: PeerJ. 2022 Oct 6;10:e13927. doi: 10.7717/peerj.13927 (PMC9548316; doi:10.7717/peerj.13927)

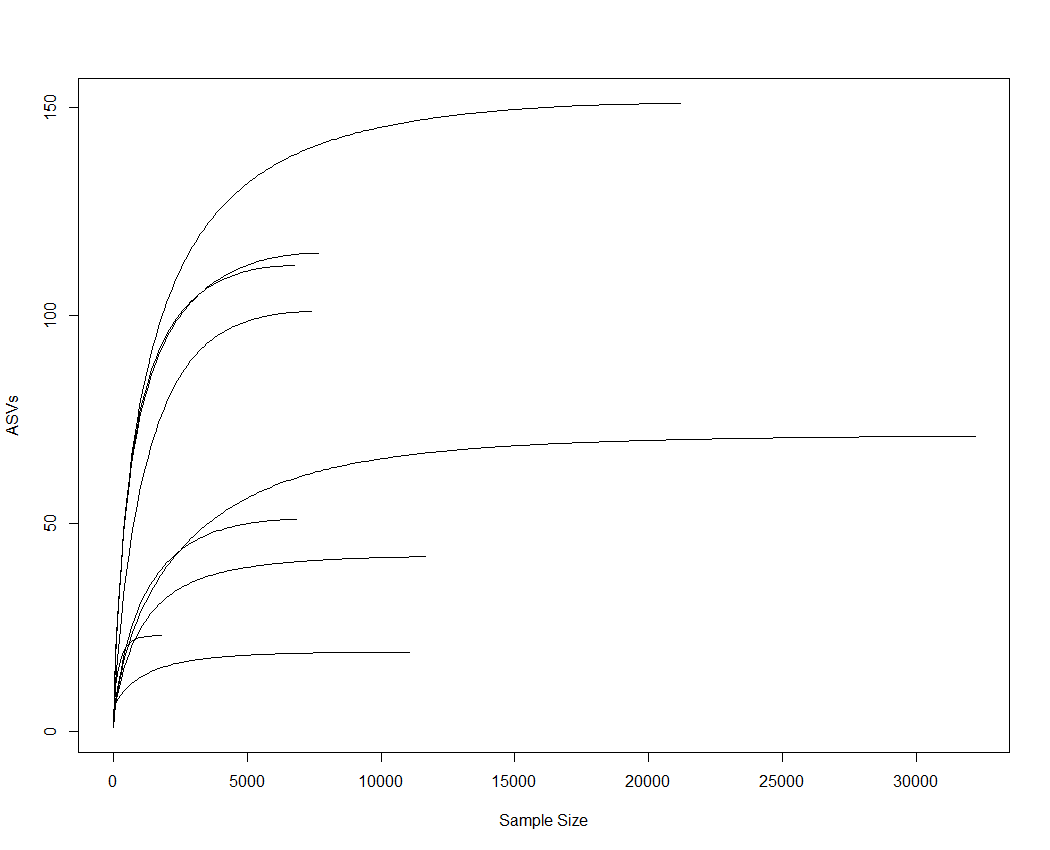

Supplement: Supplemental Information 1 [file peerj-10-13927-s001.png]

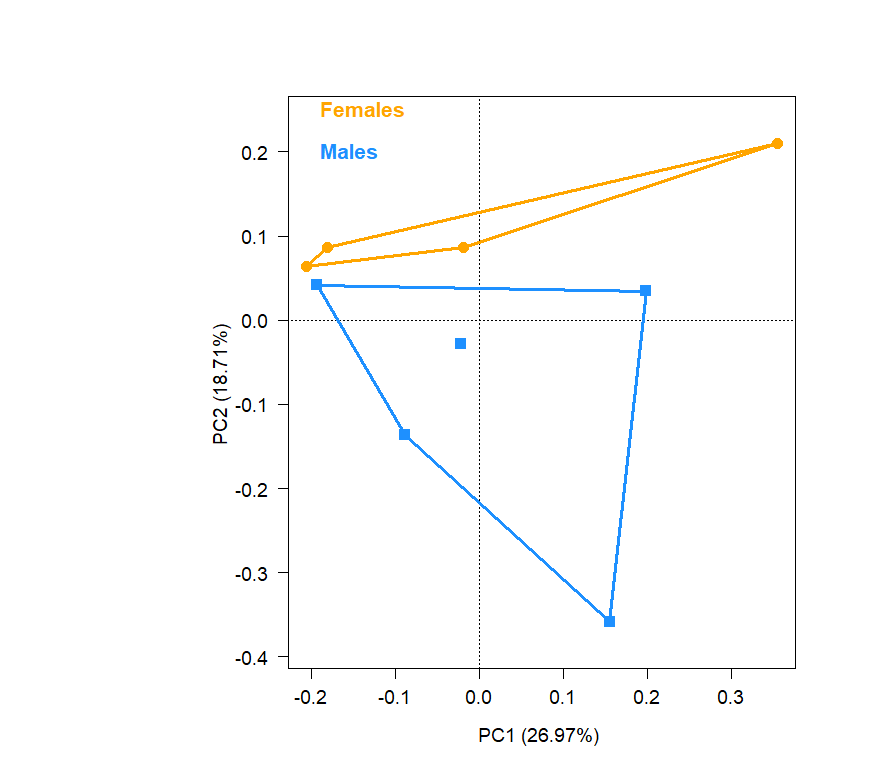

Supplement: Supplemental Information 2 [file peerj-10-13927-s002.png]
