# Supplementary material for: The cloacal microbiome of a cavity-nesting raptor, the lesser kestrel (Falco naumanni)
Source: PeerJ. 2022 Oct 6;10:e13927. doi: 10.7717/peerj.13927 (PMC9548316; doi:10.7717/peerj.13927)
Supplement: Supplemental Information 4 [file peerj-10-13927-s004.zip › code and pipeline/codes_pipeline.docx]

**Bioinformatics pipeline**

- The general DADA2 pipeline (Callahan BJ, McMurdie PJ, Rosen MJ, Han AW, Johnson AJA, Holmes SP. 2016. DADA2: High-resolution sample inference from Illumina amplicon data. *Nature methods* 13:581–583) was applied in Linux environment through R script *dada2_180719_big.data_wd.R* to infer ASVs.
- The output, saved as tab-delimited .txt, was taxonomically re-classified with the stand-alone version of the RDP classifier through the script *rdp_dada2.sh*, which contains the two nested scripts *asv_rdp_merge.py* and *asv_sort.py*, to be copied in a directory /usr/local/scripts.
